# Applications of hydrogen deuterium exchange (HDX) for the characterization of conformational dynamics in light-activated photoreceptors

**DOI:** 10.3389/fmolb.2015.00033

**Published:** 2015-06-23

**Authors:** Robert Lindner, Udo Heintz, Andreas Winkler

**Affiliations:** ^1^Department of Biomolecular Mechanisms, Max Planck Institute for Medical ResearchHeidelberg, Germany; ^2^Institute of Biochemistry, Graz University of TechnologyGraz, Austria

**Keywords:** BLUF, LOV, PYP, mass spectrometry, light sensor, effector, optogenetics

## Abstract

Rational design of optogenetic tools is inherently linked to the understanding of photoreceptor function. Structural analysis of elements involved in signal integration in individual sensor domains provides an initial idea of their mode of operation, but understanding how local structural rearrangements eventually affect signal transmission to output domains requires inclusion of the effector regions in the characterization. However, the dynamic nature of these assemblies renders their structural analysis challenging and therefore a combination of high- and low-resolution techniques is required to appreciate functional aspects of photoreceptors. This review focuses on the potential of hydrogen-deuterium exchange coupled to mass spectrometry (HDX-MS) for complementing the structural characterization of photoreceptors. In this respect, the ability of HDX-MS to provide information on conformational dynamics and the possibility to address multiple functionally relevant states in solution render this methodology ideally suitable. We highlight recent examples demonstrating the potential of HDX-MS and discuss how these results can help to improve existing optogenetic systems or guide the design of novel optogenetic tools.

## Introduction

One prerequisite for rational modulation of biological assemblies or machines is their detailed structural characterization together with an appreciation of their inherent conformational dynamics. Major contributions toward improving our understanding of molecular and cellular function have been provided by X-ray crystallography, nuclear magnetic resonance (NMR) and electron microscopy (EM). However, in isolation these methods might not suffice to understand complex multi-protein assemblies or highly dynamic signaling complexes. To overcome this limitation a plethora of lower resolution techniques can be combined with high-resolution methods to create testable models of complex biomolecular assemblies. Such approaches, summarized under the term “integrative structural biology” [1, 2], are becoming the rule rather than the exception in the functional characterization of complex systems.

In the context of optogenetic tools that allow precise spatial and temporal control of cellular processes in living cells, one generally attempts to exploit intrinsic properties of naturally occurring photoreceptors. On the one hand, the light-dependent affinity of photoreceptor proteins to different interaction partners can be employed to control localization or dimerization of various effector proteins [3–10]. On the other hand, allosteric regulation of enzymatic functions or conformational substates by photoreceptor domains provides an interesting alternative due to the potentially more direct manipulation of cellular targets [11–19]. Some optogenetic tools make use of naturally occurring designs, but substantial effort has been made to expand the optogenetic toolbox with artificial light-responsive systems [20]. Although properties of individual photoreceptors have been studied extensively, a comprehensive description of how complex light-regulated proteins work on a molecular level is frequently missing.

Structural insight into molecular mechanisms of signal transduction in light-activated enzymes can provide important molecular details for guiding the rational design of novel light-regulated systems or improving existing optogenetic tools. Due to the inherent dynamic nature of photoreceptors, their structural characterization is frequently not straight-forward, especially in the presence of corresponding interaction partners or effector domains. Since many of the functionally relevant assemblies are beyond the current size limitations of standard NMR methodologies, additional in-solution methods are required to increase our understanding of structural dynamics in photoreceptor-effector interactions. Solution scattering studies, such as small- and wide-angle X-ray scattering, of dark- and light-activated states can provide important low-resolution information that is useful to estimate the extent of structural changes accompanying light activation. In addition, many other methods can be combined with high-resolution structural characterization of photoreceptors to improve our understanding of the underlying molecular mechanisms such as for example mutagenesis [21], limited proteolysis [22], chemical crosslinking [23], EM [24], analytical spectroscopy [25, 26], light scattering [27], atomic force microscopy [28] and others.

In this review we focus on an additional powerful method for addressing molecular details of light activation: hydrogen-deuterium exchange coupled to mass spectrometry (HDX-MS). Recent developments in mass spectrometry instrumentation and automated data processing have enabled the widespread application of HDX-MS to all areas of biomedical research [29]. Here we summarize recent examples of HDX-MS for extending the structural characterization of naturally occurring photoreceptors and synthetic optogenetic tools. We also highlight how the design of optogenetic tools can benefit from such integrative structural biology approaches.

## The potential of HDX-MS

Already during the early days of HDX in the 1960s, the capabilities of quantifying protein hydrogen exchange for improving our understanding of protein structure were appreciated [30]. According to the Linderstrøm-Lang model, amide hydrogen exchange requires opening-closing fluctuations of the protein that enable protected amide hydrogens to transition to an exchange-competent state for reacting with the solvent (Scheme 1 with *k_op_* and *k_cl_* corresponding to the rate constants of the opening and closing transitions, respectively. *k_ch_* refers to the rate constant of the chemical exchange reaction of an amide hydrogen in the presence D_2_O).

$${\text{NH}}_{closed}\overset{k_{op}}{\underset{k_{cl}}{⇌}}{\text{NH}}_{open}\overset{k_{ch}}{\underset{\left({\text{D}}_{\text{2}}\text{O}\right)}{\to }}{\text{ND}}_{open}\overset{k_{cl}}{\underset{k_{op}}{⇌}}{\text{ND}}_{closed}$$

The major factor limiting hydrogen exchange is the involvement of amides in hydrogen bonding, however, additional contributions such as solvent accessibility and electrostatic effects can also influence the observed exchange rates. Since amide hydrogens play a key role in the formation of secondary and tertiary structure elements, measurements of their exchange rates can be interpreted in terms of the conformational dynamics of individual higher-order structural elements as well as overall protein dynamics and stability. Challenges associated with measuring heavy hydrogen isotope incorporation, analyzing the data generated during typical HDX experiments and interpreting the obtained information in terms of protein structure and dynamics have initially hampered the wide application of HDX [31, 32]. The development of multi-dimensional NMR methods eventually allowed measurements of site specific amide proton exchange rates. This enabled the extraction of thermodynamic parameters that reflect the equilibrium between open and closed conformations in the native state as well as the kinetic characterization of protein folding pathways [33]. Real-time, native state NMR measurements have been applied to study secondary and tertiary structure stability and to identify amides involved in strong hydrogen bonds. Due to the limited time-resolution, conventional NMR readout of hydrogen exchange generally only provides information on highly protected amides that are typically observed in regions with stable secondary structure rather than dynamic signaling elements. However, changes in physical parameters that influence deuterium exchange (for example pH and temperature) can be used to slow down the exchange reaction and provide information on otherwise inaccessible amides. Also, improvements in NMR acquisition strategies and the use of rapid mixing devices for pulsed-quench HDX enable the measurement of hydrogen exchange in the ms to s regime [34]. Still, the requirement of high protein concentrations and incomplete assignments of amide hydrogens even for small proteins also limit the potential of HDX-NMR. Recent advances in mass spectrometric analysis of deuterium incorporation have enabled the characterization of larger proteins and complex supramolecular assemblies while at the same time monitoring exchange times on the order of seconds and less, which enables sampling of highly dynamic amides. Together with the development of automated data analysis tools, the requirement of relatively low protein quantities and the possibility to work at near physiological concentrations, these improvements have resulted in a renaissance of the HDX-MS methodology over the last two decades. Currently, applications of the method span diverse areas ranging from analysis of protein interactions, characterization of individual protein conformations, protein folding studies, analysis of membrane proteins to research related to drug design [29, 35].

**Figure 1:**
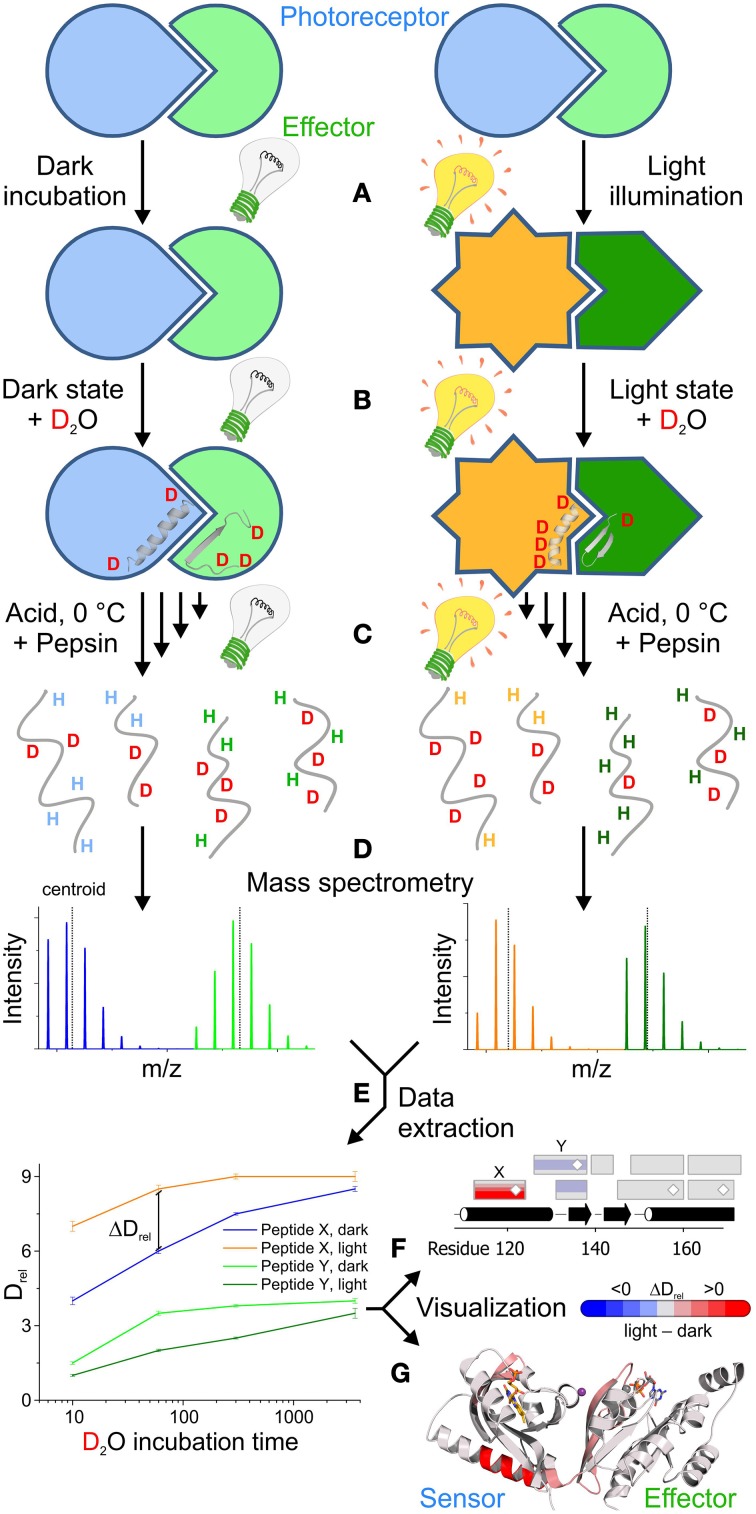
**Outline of a comparative HDX-MS experiment for the characterization of light activation in a photoreceptor-effector system**. **(A)** Typically, the experiment starts with a pre-equilibration step, to obtain a well-defined population of the dark- as well as the light-state. **(B)** Dilution of the samples with deuterated buffer initiates the substitution of exchangeable hydrogens with deuterons. The exchange rate of specific amide hydrogens reflects the conformational dynamics of their corresponding secondary structure elements in the dark- and light-state conformation. **(C)** In order to address the rate of amide hydrogen exchange, the labeling reaction is stopped at different time points by quenching with buffer at pH 2.5 and lowering the temperature to 0°C. In order to quantify deuterium uptake at a higher spatial resolution than on the global protein level, the samples are generally treated with acid-tolerant proteases, for example pepsin, to obtain individual peptides that are subsequently separated in a liquid chromatography step and then directly infused into a mass spectrometer. **(D)** The characteristic increase in peptide mass due to the substitution of hydrogen with deuterium can be readily quantified using a mass spectrometer. Due to random back-exchange of deuterons during the proteolysis and chromatography steps in aqueous media, unusually broad isotope distributions are observed for individual peptides. **(E)** Both the center of mass (centroid) and the characteristic isotope distribution are then extracted either manually or using automated software packages to identify regions with characteristic differences in deuterium uptake between the dark- and light-state. The increase of the mass centroid relative to an undeuterated reference measurement (denoted as D_rel_) for all deuteration time points provides characteristic deuterium uptake curves that enable the identification of peptides with significant differences in D_rel_ between two functional states at various time points. In combination with the assignment to a specific peptide sequence these differences in relative deuteration levels (ΔD_rel_) can be used for different visualization approaches that also provide information on overlapping peptides and corresponding secondary structure elements **(F)** or the three dimensional arrangement of the individual peptides **(G)**. In this scenario, blue colors correspond to less deuterium being incorporated in the light state due to a reduction of the conformational dynamics. In contrast, red colors reflect increased deuterium incorporation at a given time point corresponding to an increase in structural dynamics upon illumination. Results shown in **(E,F)** are not linked to **(G)**, which shows the photoreceptor architecture and HDX-MS results of PA-Rac1 [83] that are described in more detail in **Figure 5**.

One reason for the broad application of HDX-MS is its ability to provide information on the global stability as well as on local conformational dynamics of proteins. Since proteins are dynamic entities that exhibit local and global conformational fluctuations, even the most stable secondary structure elements transiently break and re-form hydrogen bonds. According to the opening-closing model mentioned previously, deuterium exchange can only occur while loss of structural elements exposes hydrogens to the solvent. Depending on the rate constants of the opening (*k_op_*) and closing (*k_cl_*) reaction as well as the rate constant of chemical exchange (*k_ch_*) under the specific conditions of the experiment, characteristic time dependent changes in the isotope distribution of individual peptides can be observed. An in depth discussion of the underlying mechanisms is beyond the scope of this article, however, details can be found elsewhere [31, 33, 36, 37]. Briefly, under conditions where *k_cl_* is significantly faster than *k_ch_*, there is only a low probability that a single opening event will lead to hydrogen exchange. This regime, termed EX2, is most frequently observed under native conditions and is characterized by a time-dependent, gradual increase of the isotopic distributions of individual peptides measured in a typical HDX-MS experiment (*cf*. Figure 1). The observed rate constant of hydrogen exchange (*k_ex_*) under EX2 conditions reflects the thermodynamic equilibrium constant of closed and open states (*K_op_* = *k_op_*/*k_cl_*) according to the expression *k_ex_* ≈ *K_op_ · k_ch_*. The other extreme, where *k_ch_* ≫ *k_cl_*, is termed EX1 and corresponds to the situation where every opening event is accompanied by chemical exchange. The observed rate constant *k_ex_* under EX1 conditions therefore directly corresponds to the structural opening rate constant *k_op_* (*k_ex_* ≈ *k_op_*) providing direct access to kinetic data. Provided that several amide positions within a peptide follow the EX1 exchange, characteristic bimodal isotope distributions are observed, which are also referred to as correlated exchange patterns. Even though no clear distinction between sub-global unfolding or local fluctuations can be made based on the measured deuterium exchange rates of peptides, the ability to measure the time-dependent increase in deuterium incorporation and the characteristic peak shape of the isotopic distributions provides insight into the conformational dynamics of the studied system. Since dynamic properties of a system are tightly linked to protein function [38], it is obvious that a combination of HDX with crystallographic snapshots can substantially improve the understanding of system specific functions.

Another reason for the success of the methodology is the information content of comparative HDX experiments. Since the labeling reaction can be performed at low protein concentrations in solution, many different functionally relevant states, that might be difficult to address with other methods, can be characterized using HDX-MS. Although rich in information content, comparative analysis of multiple functional states entails increasing combinatorial complexity in data analysis; hence, development of automated data extraction and analysis software packages was essential to exploit the full potential of HDX-MS.

## Typical workflow for HDX-MS characterization of a photoreceptor

Due to the diversity of applications, different protocols for effectively using HDX-MS have been developed over the years [summarized in Konermann et al. [37]]. The most commonly used technique is “continuous labeling” where the native protein is exposed to deuterated buffer solutions and the incorporation of the deuterium label is monitored over time. A schematic workflow of this approach geared toward the characterization of a photoreceptor in its dark- and light-state is illustrated in Figure 1. In order to measure exchange characteristics representing the conformation of interest, it is important to pre-incubate the samples under conditions that provide high percentages of the protein in the respective state prior to the dilution in deuteration buffer (Figure 1A). Depending on the nature of the photoreceptor system, bi-stable light sensors might have to be switched to a specific functional state using appropriate light sources, whereas photoreceptors that exhibit a thermal dark state recovery can be incubated sufficiently long in the dark to ensure full population of the dark state. In order to obtain a significantly light-state biased steady-state population of the photoreceptor for the comparative measurement, the length of the pre-illumination step and the light intensity need to be adjusted depending on the rate of dark state recovery.

Dilution in D_2_O-containing buffer then triggers hydrogen exchange under appropriate light conditions that maintain the steady state population of the dark- or light-state conformations (Figure 1B). In typical HDX-MS experiments hydrogen exchange is stopped after various time points ranging from seconds to hours and sometimes days by quenching the reaction in low pH buffer and lowering the temperature to 0°C (Figure 1C). In the case of photoreceptors, control experiments need to be performed to rule out any side reactions, such as cofactor bleaching, during prolonged incubation times; depending on the employed light intensities such effects generally limit the accessible time range to a few hours. Since the chemical exchange rate of amide hydrogens is slowest around pH 2.5 and due to the temperature dependence of any chemical reaction, the conditions used for quenching of the reaction, on the one hand, minimize the loss of deuterium label during the subsequent readout procedure in aqueous environment (termed back-exchange) and, on the other hand, favor the loss of deuterium incorporated at other exchangeable positions in polar side chains [33].

The increase in mass due to deuterium incorporation measured in the mass spectrometer is determined by the exchange characteristics of the backbone amide positions. While direct mass spectrometric analysis of the full-length protein after desalting can be used to estimate global changes in protein stability and dynamics upon illumination, more detailed information on how specific structural elements are affected can be obtained by proteolytic digestion. Immobilized acid tolerant proteases, such as pepsin, can be integrated into an automated liquid chromatography setup enabling rapid digestion, desalting and separation of the peptides prior to infusion into the mass spectrometer (Figure 1D). Due to the relatively unspecific cleavage properties of acid tolerant proteases, many overlapping peptides with variable abundance are measured during the mass spectrometric analysis. In combination with the unusually broad individual isotope distributions, due to random back-exchange events, this results in complex spectra that need to be evaluated.

Since manual data extraction and analysis is a tedious, time consuming task, automated software packages are generally used to extract the relative shift in mass centroids for given peptides in multiple time points and repetitive measurements (Figure 1E). As discussed previously, also the shape of the isotope distribution can provide important insight into molecular aspects of the exchange reaction and should be extracted for data analysis. Eventually, individual peptides need to be assigned to a specific sequence of the protein and can then be compared between different functional states. Characteristic deuterium uptake plots showing the time dependent increase in deuteration level relative to an undeuterated reference (D_rel_, Figure 1E) provide information on the overall protection of structural elements and their conformational dynamics. More localized information might be obtained by correlating all peptides with the primary and secondary structure of the protein (Figure 1F) and extracting the information content available from overlapping peptides. If available, mapping onto the three-dimensional structure (Figure 1G) can ultimately help to identify potential signaling pathways due to the identification of structural elements that are affected by illumination in both the photoreceptor and effector domains. Since most of the routines required during data extraction and visualization are repetitive processes, automation of this workflow greatly helps to reduce the time required to interpret deuterium exchange experiments. In this context, the HDX-MS community benefits from the standardized instrumentation and data formats as well as from powerful algorithms for signal processing, feature detection and peptide assignment [39, 40] originating from the widespread adaption of proteomics research.

## Improvements in HDX-MS data evaluation

A major challenge in HDX data analysis is to identify and match signals originating from peptide species with unknown H/D compositions, and at the same time, to quantify the abundance of each deuterated species. A number of computational solutions have been proposed for individual parts of the HDX analysis workflow, for example quantification of deuterium incorporation [41–44], chromatogram alignment [45], or statistical evaluation of deuteration differences between different samples [46]. Hydra [47] was the first solution capable of providing a full analysis workflow and a number of web server based programs followed [48, 49]. ExMS [50] is a suite of MATLAB scripts which offer visualization for interactive parameterization. HDX-Workbench [51] is geared toward high-resolution HDX-MS data and offers a comprehensive graphical interface with visualization and manipulation capabilities. Hexicon 2 [52] adapted and extended the regularized feature detection and deuteration distribution estimation algorithms from its predecessor [53] for improved performance with high-resolution data. This recent generation of data analysis tools provides solutions for most data processing needs of a bottom-up HDX-MS experiment as described above and features rich graphical access to data, thus enabling on-line quality control and fast generation of conclusive results. Another advantage of modern software packages is their ability to identify and quantify differently deuterated subpopulations of individual peptides. A good estimate of the deuteration distribution provides insight into the dynamics of multi-state equilibria which may otherwise not be identified as such [54]. For some of the photoreceptor characterizations discussed below, where illumination modulates the equilibrium between dark- and light-states, robust deuteration distribution estimation was essential for valid interpretation of HDX-MS data.

The ease of data processing empowered the community to develop novel experimental protocols, for example, using electron transfer dissociation for resolution enhancement [55] or protease-free top-down fragmentation [56, 57]. Besides the continuous improvement of data processing algorithms, advancements in experimental technology and new applications of HDX-MS call for flexible solutions that let the user match data analysis to the experimental strategy. One development in this direction is Mass Spec Studio [58], featuring a modular architecture that allows users and developers to reuse core components and to tailor the workflow to their needs.

## Challenges in the characterization of light-sensitive proteins

All photosensory modules used in optogenetics originate from natural photoreceptors that typically employ small molecule cofactors to sense light in specific regions of the electromagnetic spectrum. Cofactor-binding photoreceptors can be classified into six different families: Retinal binding rhodopsins [59], *p*-hydroxycinnamic acid binding xanthopsins [60], bilin binding phytochromes [61], as well as the group of flavin containing photoreceptors [23, 62] such as cryptochromes, light-oxygen-voltage sensing (LOV) domain containing proteins and sensor of blue light using FAD (BLUF) domain containing proteins. In addition to cofactor-binding photoreceptors, a UV-B sensitive plant photoreceptor termed UV resistance locus 8 (UVR8) has been shown to use tryptophan residues for light perception [63]. In all cases, photon absorption induces local structural changes near the cofactor which are then coupled to rearrangements of intra- or inter-protein contacts that result in channel opening, ion pump activation, regulation of various effector domain activities, or modulation of protein-protein interactions.

**Figure 2:**
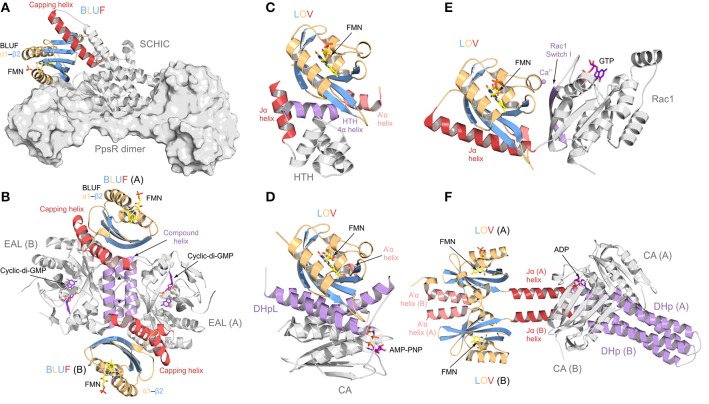
**Protein architectures of full length BLUF and LOV photoreceptor-effector systems**. Photoreceptor domains are colored according to secondary structure: helices, orange; β-sheets, blue. N- and C-terminal helical domain extensions are colored in light and dark red, respectively, and elements important for signal transduction are colored in purple. **(A)** AppA-PpsR system from *Rhodobacter sphaeroides* [27]. AppA (shown as cartoon) senses blue light using a BLUF domain and, via its C-terminal SCHIC domain, binds to a dimer of the transcriptional repressor PpsR (a truncated construct is shown in surface representation). Illumination alters the DNA-binding ability of PpsR through allosteric modulation of its helix-turn-helix motif (HTH; not present in the AppA-PpsR_2_ core complex structure—*cf*. **Figure 6**). **(B)** Blue-light regulated phosphodiesterase 1 (BlrP1) from *Klebsiella pneumonia* [86]. BlrP1 contains an N-terminal BLUF domain and a C-terminal EAL phosphodiesterase domain. The protein homo-dimerizes through a conserved interface made up of two dimerization helices and one compound helix formed by two short helices provided by each protomer (both shown in purple). Communication between the BLUF domains and the EAL active sites is bidirectional and mediated through the dimer interface [87]. **(C)** Light-activated transcription factor EL222 from *Erythrobacter litoralis* [14]. In contrast to the well-studied LOV2 from *Avena sativa* phototropin (shown in **(E)** as part of PA-Rac1), the C-terminal Jα helix (shown in red) does not pack against the LOV β-sheet. Instead it is replaced by a helix of the HTH DNA-binding motif. Illumination releases the HTH motif from the LOV β-sheet and thus enables DNA binding. **(D)** Light-activated monomeric histidine kinase EL346 from *Erythrobacter litoralis* [22]. Two α-helices of a dimerization/histidine phosphotransfer-like (DHpL) domain (shown in purple) pack against the LOV β-sheet and couple the photoreceptor to the catalytic/ATP-binding (CA) domain with bound adenosine 5′-(β, γ-imido)triphosphate (AMP-PNP, shown as stick model). Illumination is assumed to loosen the LOV-DHpL interactions, thus enabling CA domain activation. **(E)** Synthetic photoactivatable GTPase Rac1 (PA-Rac1) [19]. PA-Rac1 was generated by fusing the LOV2 domain from *Avena sativa* phototropin 1 to the small human Rac1 GTPase. A Ca^2+^ ion (purple sphere) bound in the interdomain interface restricts the dynamics of the Jα helix (dark red) packed against the LOV domain core. Illumination perturbs the Jα helical structure and renders the interface more dynamic, allowing the GTPase to engage in β-strand pairing with downstream effectors through its peripheral β-strand (purple) [83]. **(F)** Synthetic light-regulated histidine kinase YF1 [93]. The dimeric protein was generated by replacing the oxygen-sensing PAS domain of *Bradyrhizobium japonicum* FixL with structurally homologous LOV photoreceptor domain from *Bacillus subtilis* YtvA. The main part of the LOV dimer interface is formed by the N-terminal flanking helices (light red) that pack against the LOV β-sheet of the opposite subunit of the dimer. The C-terminal Jα helices (dark red) form a coiled coil structure extending away from the photoreceptor domains, connecting them to a dimerization/histidine phosphotransfer (DHp) domain (colored in purple) which forms an interaction platform for the CA domains. The activation mechanism is proposed to involve twisting of Jα upon illumination.

Structural studies of individual members of these photoreceptor families using X-ray crystallography have significantly contributed to a better understanding of molecular aspects of light-signaling. A variety of crystal structures of isolated photoreceptor domains in their dark states have been published, however, in many cases these structures are lacking the light-regulated output domains. In order to better appreciate potential signaling pathways to diverse effector domains and to identify similarities or differences in various members of the photoreceptor families, more crystal structures of full-length photoreceptors or light sensors in complex with their non-covalently linked effector domains would be helpful. In this respect, significant progress was recently made predominantly in the field of flavin containing BLUF and LOV proteins. Representative architectures are summarized in Figure 2 and their comparison reveals intriguing insight into the coupling of sensors and effectors. Individual members of both families have very similar core photoreceptor structures, however, the N- and C-terminal extensions show characteristic differences between various systems. As illustrated in Figure 2, these flanking regions adopt different architectures tailored to the requirements of the specific effector system. From this it appears difficult to generalize signaling mechanisms even within a family of photoreceptors and additional functional studies addressing the coupling of sensor and effector will be required to better understand functioning of complex photoreceptors.

**Figure 3:**
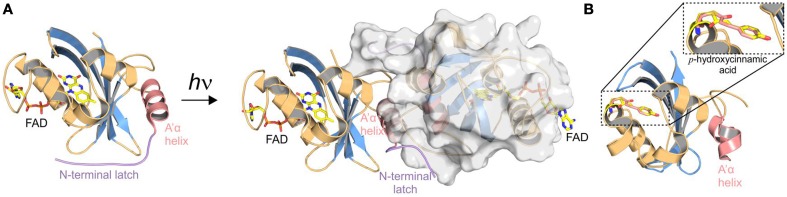
**Selected structures obtained from crystals grown in the dark and light-activated state vs.** ***in crystallo*** **light-activated photoreceptors**. The PAS/LOV domains are shown in similar orientation and are colored according to Figure 2. **(A)** Crystal structures of dark- [76] and light-adapted [64] VIVID from *Neurospora crassa*. Blue light illumination of VIVID changes the oligomeric state from monomers to dimers. Dimerization is mediated by release of the N-terminal latch (shown in purple) from the protein core, enabling interaction with the acceptor pocket of another protomer. **(B)** PYP from *Halorhodospira halophile* in its light activated conformation [72]. Only one representative structure is shown, since no global structural rearrangements are observed upon illumination of dark-grown crystals. Modest structural changes that affect the hydrogen-bonding network around the cofactor are observed upon light activation of PYP. Illumination triggers a *trans*-*cis* isomerization of the *p*-hydroxycinnamic acid cofactor (yellow and light-red, respectively) that is covalently bound to a cysteine residue.

Another important aspect of the structural analysis of photoreceptors is the characterization of light-activated states. However, trapping photoreceptors in their light state conformation is a major obstacle for crystallographic studies and only few structures of crystals grown in the light-activated state are available. So far this has only been achieved for the single domain LOV proteins VIVID (VVD) [64] (Figure 3A) and PpsB1 [65] as well as for a truncated phytochrome construct [66], all of which revert slowly into their dark state conformation. For most photoreceptors, the increase in structural dynamics upon illumination makes crystallization and also NMR studies difficult. To circumvent the problems linked to the structural characterization of short-lived and highly dynamic light states, dark state crystals have been illuminated to study light-induced conformational changes [26, 67–76]. One of the best characterized photoreceptors using this approach is the xanthopsin member PYP that is shown for illustration purposes in Figure 3B. Although illumination of dark-grown crystals provides insight into local structural rearrangements upon light activation, crystal lattice constraints prevent larger conformational changes and thus limit the analytical power of this technique. However, to understand signal propagation in photoreceptors on a structural and functional level, a detailed characterization of all functionally relevant signaling states is desirable. In this respect, HDX-MS is a powerful supplementary method for identifying structural elements affected by illumination as well as for the characterization of multiple functionally relevant photoreceptor states.

## HDX-MS applications in the field of photoreceptors

**Figure 4:**
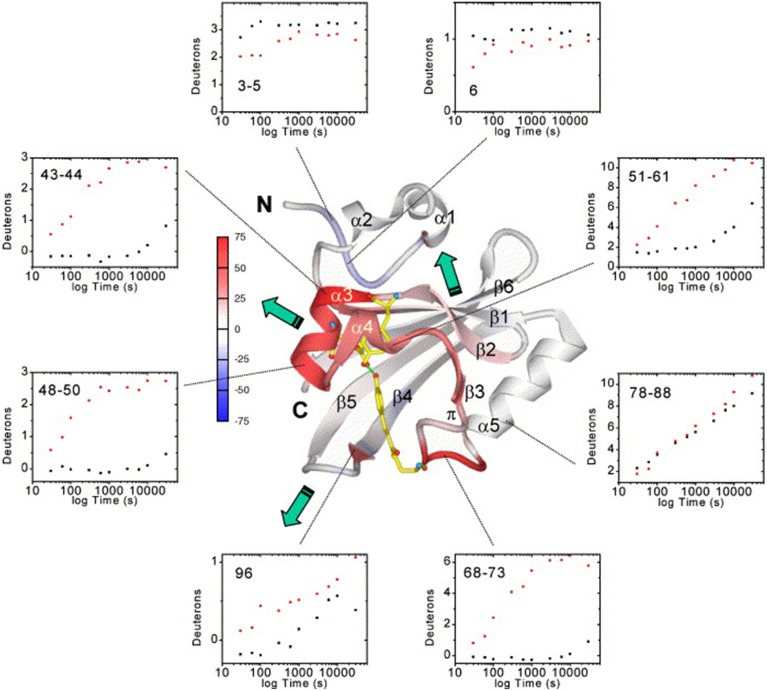
**Average deuteration changes upon illumination mapped on the PYP structure, H/D exchange kinetics and signal transduction pathways [77]**. Average deuterium incorporation changes are mapped on the PYP dark-state ribbon diagram colored from −75 to +75% change according to the color bar. Secondary structure elements and the N- and C- termini are labeled. The chromophore and active-site residues Tyr42 and Glu46, which form hydrogen bonds (green dotted lines) to the phenolate oxygen atom of the chromophore in the dark state, are shown for better orientation. Green arrows indicate the three signal transduction pathways (through the PAS core helices, the β-hairpin, or the β-sheet interface). H/D exchange kinetics (black dots, dark state; red dots, I2 light state) are shown as examples of the data quality and the time-course of H/D exchange in representative PYP sequence segments. Reprinted from Brudler et al. [77] Copyright © 2006, with permission from Elsevier.

In the last decade, several studies have been performed that demonstrate the applicability of HDX-MS for the functional characterization of photoreceptors. The strategy common to most of these experiments is continuous labeling of the investigated proteins in the dark and under constant illumination, followed by bottom-up LC-MS analysis (*cf*. Figure 1). Photoactive Yellow Protein (PYP) from *Halorhodospira halophila* is the prototype of the xanthopsin protein family and was the first photoreceptor to be studied using HDX-MS [77, 78]. PYP exhibits a PAS fold which comprises a five-stranded antiparallel β-sheet surrounded by α-helices and covalently binds a *p*-hydroxycinnamic acid cofactor via a cysteine residue. Upon blue light illumination, the cofactor undergoes a *trans-cis* isomerization that changes the hydrogen bond network around the ligand and induces receptor activation (Figure 3B). Structural differences between the dark- and light-states of PYP were localized to the direct vicinity of the *p*-hydroxycinnamic acid chromophore, where several residues form a characteristic hydrogen bond network around the *trans*-chromophore in the dark. HDX studies confirmed that photoisomerization of the chromophore destabilizes these interactions, as reflected in increased deuterium uptake and therefore increased conformational dynamics of elements flanking the chromophore binding site [77, 79]. While HDX-NMR experiments only identified elements in the direct vicinity of the cofactor [79], HDX-MS measurements revealed the propagation of the allosteric signal to helices flanking the PAS core and also to the β–sheet and the N-terminal capping region as shown in Figure 4 [77].

Additional PAS photoreceptors that were studied by HDX belong to the family of LOV domains that, in contrast to PYP, contain a non-covalently bound flavin cofactor (FMN or FAD). In most LOV domains, the globular core is flanked by α-helical N- and C-terminal extensions that pack against the surface of the LOV β-sheet and play an important role in signal transduction [23]. Upon illumination with blue light, the flavin cofactor forms an adduct with a conserved cysteine residue, which initiates a cascade of structural changes propagating from the chromophore to the domain boundaries. Continuous labeling HDX-MS studies on the LOV domain protein VIVID that was shown to homodimerize in a light-dependent manner [76] showed that the intact photoreceptor reversibly unfolds and that the rate of unfolding decreases upon illumination [80]. Experiments on the peptide level revealed that stabilization of the PAS β-sheet and the central helical region close to the flavin cofactor explain the reduced unfolding upon illumination. In contrast, the high exchange rates of the N-terminal flanking helix that plays an important role in light induced dimerization [64], were hardly affected by illumination. Moderate protection of the crystallographic dimer interface upon illumination is in line with a rapid monomer-dimer interconversion [81]. These observations contrast with the above described studies on PYP, where illumination caused significantly increased amide proton exchange of helical PAS regions and slightly protected the N-terminus [77]. Therefore, different interactions of the flavin and the *p*-hydroxycinnamic acid chromophores as well as differences in the oligomerization state result in contrary effects with respect to conformational dynamics of two PAS-sensors and suggest that PAS signaling can operate via diverse mechanisms.

Even within the family of LOV photoreceptors, diverse effects on protein dynamics are observed in the lit state. NMR studies on the isolated LOV2 domain from *Avena sativa* phototropin 1 showed destabilization and undocking of the C-terminal helical extension (Jα) from the LOV β-sheet upon illumination [82]. In the isolated domain, the Jα helix is highly dynamic already in the dark such that deuterium exchange was too rapid to capture any differences between dark and lit states using either HDX-NMR or -MS methods [82, 83]. In contrast to VIVID, which does not contain a C-terminal helical extension, the core LOV2 domain showed no transient unfolding in HDX-MS experiments and only structural elements surrounding the flavin cofactor were affected by illumination [83]. Interestingly, HDX-NMR studies with isolated LOV2 indicate that the light state is destabilized sufficiently to undock the Jα helix without globally unfolding the PAS core [82]. Another remarkably different member of the LOV family that is small enough to be studied by NMR is the light-regulated transcription factor EL222 from *Erythrobacter litoralis*. It comprises an N-terminal LOV domain fused to a DNA-binding helix-turn-helix motif of which one helix is docked against the surface of the LOV β-sheet (Figure 2C) in a configuration resembling the C-terminal Jα helix of LOV2. A combination of HDX-NMR and chemical shift difference analysis showed that the helix undocks from the LOV domain upon illumination but at the same time remains protected from exchange on NMR timescales, indicating that illumination releases the domains from each other without perturbing any stable secondary structures [14].

**Figure 5:**
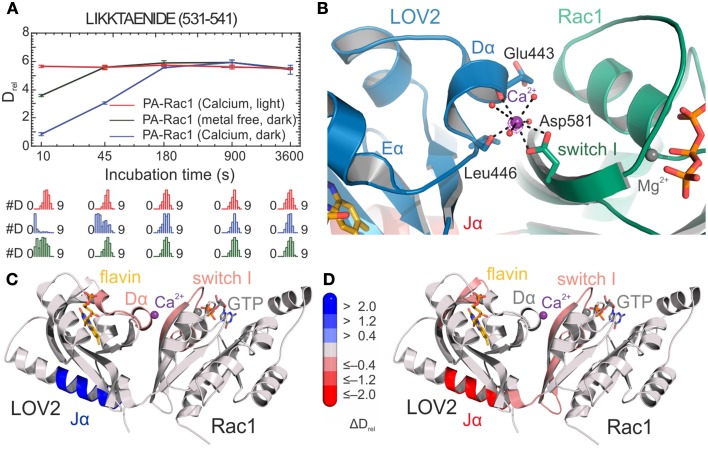
**HDX-results of the optogenetic tool PA-Rac1 [83]**. **(A)** Deuterium uptake curves of a representative LOV2-Jα peptide with relative deuteration values (*D*_rel_) plotted against deuteration time for three different functionally relevant states. *D_rel_* values are shown as the mean of three independent measurements, and error bars correspond to the standard deviation. Lower panels show the Hexicon 2 estimated abundance distribution of individual deuterated species on a scale from undeuterated to all exchangeable amides deuterated [52]. The bimodal distributions indicate distinct conformational states of the Jα helix. **(B)** Calcium binding site at the LOV2–Rac1 interface. Coordinating residues from both LOV2 and the Rac1 domain providing four of the eight oxygen ligands are shown as stick models. The remaining water ligands are represented as spheres. **(C,D)** Structures of PA-Rac1 colored according to the differences in relative deuteration levels upon calcium coordination in the dark and illumination in the presence of calcium ions, respectively. Coloring corresponds to the differences in relative deuteration of individual peptides according to the bar legend [52] and highlights the effect of metal binding on the functionally relevant Jα helix and the LOV2-Rac1 interface. Shades of red or blue colors reflect an increased or decreased deuterium uptake, respectively. Reprinted with permission from Winkler et al. [83]. Copyright © 2015 American Chemical Society.

In the context of multi-domain LOV-based photoreceptors, HDX-MS was used to study the effect of illumination for the synthetic photoactivatable GTPase Rac1 (PA-Rac1) [83] that had been proposed to function by release of the caged GTPase domain from the LOV photosensor upon illumination [19] (Figure 2E). Deconvolution of isotopically resolved MS spectra revealed the existence of two distinct conformational states of the Jα helix flanking the LOV core and connecting the photoreceptor with the GTPase. Blue-light illumination shifts the relative populations of these conformational states (Figure 5A) as previously described [84]. Of note, metal coordination at an inadvertently created binding site at the interdomain interface (Figure 5B) substantially increases the dynamic range by stabilization of the caged Jα conformation only in the dark (Figures 5C,D). In combination with solution scattering experiments, HDX-MS data suggested that illumination allosterically modulates GTPase dynamics rather than releasing the GTPase. These results highlight that, in addition to the considered photoreceptor activation mechanism, molecular details of engineered sensor-effector interfaces as well as structural dynamics of the dark-light transition are relevant for the successful design of artificial photoswitches.

Similar to LOV domains, also BLUF domains are small globular photoreceptor domains that bind a flavin cofactor, and exhibit a ferredoxin-like protein fold with two helices aligned parallel to a five stranded β-sheet. As observed for LOV proteins, also BLUF domains are frequently C-terminally flanked by a helix that packs against the surface of the central β-sheet and is likely involved in signal transduction. Light-activation causes a rearrangement of the hydrogen-bonding network around the cofactor, including tautomerization of an essential glutamine residue [85], which eventually alters the dynamics of the C-terminal β-strand and the adjacent helical extension. A multi-domain BLUF protein that was studied by HDX-MS is the blue-light regulated phosphodiesterase 1 (BlrP1) from *Klebsiella pneumoniae* that features a BLUF domain covalently linked to a metal-dependent EAL phosphodiesterase (Figure 2B). BlrP1 degrades the second messenger cyclic dimeric GMP in a light-stimulated fashion [86]. HDX-MS experiments addressing substrate and metal ion binding in the dark- and light-states of the characteristic BlrP1 dimer revealed a bidirectional communication between regions involved in light sensing in the BLUF domain and regions coordinating the catalytically relevant metal ions in the EAL domain [87]. The EAL dimer interface with its characteristic compound helix was shown to function as a central communication platform for the allosteric EAL-BLUF cross-talk, which is intensified upon illumination. Combination of all analyzed BlrP1 states allowed the mapping of a signaling pathway between the sensor and effector domains of the complex multi-domain protein and provided molecular details of regions involved in inter-domain communication.

**Figure 6:**
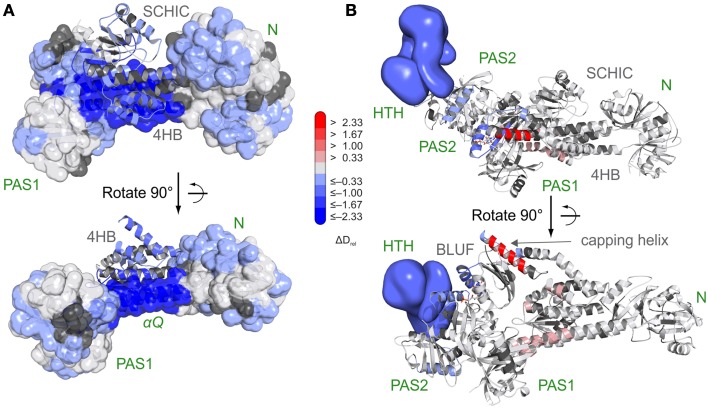
**Characterization of the AppA-PpsR_2_ complex using HDX-MS [27]. (A)** Superposition of HDX data addressing AppA-PpsR_2_ complex formation of full length PpsR and C-terminally truncated AppA (AppA_ΔC_) with the crystallized AppA-PpsR_2_core complex. HDX-MS data of the 15 s deuterium exchange time points are mapped onto the complex with the PpsR N–Q–PAS1 construct (N-terminal PAS domain—N; Q-linker—αQ; PAS1 domain) shown in transparent surface and the AppA domains (four helix bundle—4 HB; SCHIC) in cartoon representation. **(B)** Model of the AppA_ΔC_–PpsR_2_ complex as described in Winkler et al. [27] colored with respect to the observed changes in deuterium incorporation between light- and dark-state measurements of the complex. The 15 s deuterium exchange time point is shown for visualization of protection (blue) or destabilization (red) upon complex illumination. Reprinted with permission from Winkler et al. [27], Nature Publishing Group.

Due to high-resolution mass spectrometers and sophisticated data evaluation tools, even more complex systems can nowadays be studied by HDX-MS. Experiments with the AppA-PpsR photoreceptor-effector system from *Rhodobacter sphaeroides* demonstrated that even mixtures of multi-domain proteins no longer pose a challenge for data processing and sequence assignment [27]. The AppA-PpsR system, which plays a role in photosynthesis-related gene expression, forms a non-covalent AppA-PpsR_2_ heterotrimeric complex [88]. AppA is composed of a light-sensing BLUF domain and a SCHIC domain that was shown by HDX-MS analysis to be involved in complex formation with the transcriptional regulator PpsR (Figure 6A). Based on exchange data, crystallization constructs were designed and allowed structure determination of an AppA-PpsR_2_ core complex [27]. Combination of functional, crystallographic [89] and HDX-MS experiments showed that illumination of the AppA-PpsR_2_ complex influences the α1-β2 region of the AppA-BLUF domain and its C-terminal capping helix, which results in stabilization of the DNA-binding helix-turn-helix motif of PpsR and thus interferes with DNA-binding of the effector (Figure 6B). Together, the data suggest the involvement of a light-sensitive ternary AppA-PpsR-DNA complex in photosynthesis-gene regulation which had previously not been appreciated. In comparison to HDX-NMR experiments on the isolated AppA BLUF domain [90], it should be noted that the light-responsive elements identified by HDX-MS are highly dynamic with nearly complete deuterium exchange occurring within the dead time of a typical 2D NMR measurement.

Although complex soluble photoreceptor systems can be addressed by HDX-MS, the characterization of transmembrane proteins such as rhodopsin photoreceptors remains challenging. Based on their function three rhodopsin classes can be distinguished: sensory rhodopsins, ion-pumps for H^+^ and Cl^−^ as well as ion-channels. Although the different rhodopsins classes exhibit low sequence identity, they all comprise seven transmembrane helices and sense light using a retinal cofactor. The cofactor is bound to a conserved lysine forming a protonated Schiff base. Depending on the protein environment, rhodopsins can absorb photons of almost the entire visible range. Illumination results in photoisomerization of the retinal from an all-*trans* to a 13-*cis* configuration which triggers protein activation and initiates the photocycle. Bacteriorhodopsin (BR) is the simplest and one of the best studied rhodopsins and functions as a green light regulated proton pump that transports protons out of the cell. It is embedded into purple membranes where it naturally forms 2D crystals composed of BR trimers. In line with the tight packing of BR in purple membranes, HDX-MS studies showed nearly indistinguishable isotope exchange behavior under dark and light conditions [91]. Purple membranes exhibit a high degree of protection and even after extended labeling periods less than half of the amide protons of trimeric BR were exchanged. To circumvent this problem, HDX experiments on monomeric BR were performed in bicelles. Comparative experiments revealed pronounced destabilization of the protein upon illumination indicating that light-activated BR undergoes substantial structural changes [91]. Individual structural elements affected by illumination could not be identified because the presence of membranes or detergents impedes protease digestion and peptide separation and thus complicates spatially resolved bottom-up HDX-MS. In order to improve mechanistic interpretations, new and better strategies to detect deuteration of transmembrane proteins are currently being developed [29] and one such approach has recently been used in the context of photoreceptors for refolding studies with BR [92].

## Conclusion

In summary, the studies described highlight HDX-MS as a powerful method for the detailed characterization of multiple functionally relevant signaling states of photoreceptors. In combination with additional functional or structural data, HDX-MS can significantly improve our understanding of signal integration in diverse light sensor domains, help in the identification of potential signaling pathways to various effector domains and also provide information on how the biological function of diverse output domains is regulated.

As far as the characterization of PAS/LOV and BLUF domains is concerned, HDX-MS analyses reveal that signaling state formation of different sensors within a family can be accompanied by diverse modes of conformational changes. Importantly, isolated sensory modules show different conformational dynamics compared to their analysis in the presence of the regulated effector domain. Depending upon the desired mode of action of novel optogenetic tools, a more detailed characterization of full-length members of different photoreceptor families in terms of light-induced structural rearrangements will be required to enable a more rational design of optogenetic systems that employ building blocks of naturally evolved sensor-effector couples. To improve the functionality of artificial tools, ultimately, also the specific requirements for regulation of different effector domains and the conformational determinants that affect the dynamic range of the system need to be appreciated.

In the case of the established optogenetic tool PA-Rac1, HDX-MS analysis revealed that an inadvertently introduced metal ion binding site influences the dynamic range and potentially provides an explanation for the unsuccessful attempts to transfer the concept of PA-Rac1 to closely related GTPases [83]. In this respect, reengineering of the sensor-effector interfaces of similar LOV2-GTPase fusions to enable metal binding or to decrease the conformational dynamics of the corresponding effector regions by different means would be a promising rational approach to further expand the optogenetic toolbox. The observation that properties of newly generated interfaces can have pronounced effects for the functionality of optogenetic tools further emphasizes the need for a detailed characterization of existing tools to better understand their functionality *in vitro* and ultimately also *in vivo*.

Considering the variety of photoreceptor families and the modularity of naturally occurring systems with diverse effector domains, the application of HDX-MS in the field of light responsive proteins has so far only touched the tip of the iceberg. Recent developments in mass spectrometry instrumentation and data analysis automation are expected to increase the utility of HDX-MS in integrative structural biology and also in the field of photoreceptor research. This might provide interesting insight into signaling mechanisms of additional photoreceptor families such as cryptochromes and phytochromes, which are frequently employed modules in optogenetic tools.

### Conflict of interest statement

The authors declare that the research was conducted in the absence of any commercial or financial relationships that could be construed as a potential conflict of interest.
